# Bacteriophage Φ21’s receptor-binding protein evolves new functions through destabilizing mutations that generate non-genetic phenotypic heterogeneity

**DOI:** 10.1093/ve/veae049

**Published:** 2024-07-11

**Authors:** Krista R Gerbino, Joshua M Borin, Sarah M Ardell, Justin J Lee, Kevin D Corbett, Justin R Meyer

**Affiliations:** School of Biological Sciences, University of California San Diego, 9500 Gilman Dr, La Jolla, CA 92093, United States; School of Biological Sciences, University of California San Diego, 9500 Gilman Dr, La Jolla, CA 92093, United States; School of Biological Sciences, University of California San Diego, 9500 Gilman Dr, La Jolla, CA 92093, United States; School of Biological Sciences, University of California San Diego, 9500 Gilman Dr, La Jolla, CA 92093, United States; School of Biological Sciences, University of California San Diego, 9500 Gilman Dr, La Jolla, CA 92093, United States; Department of Cellular and Molecular Medicine, University of California San Diego, 9500 Gilman Dr, La Jolla, CA 92093, United States; School of Biological Sciences, University of California San Diego, 9500 Gilman Dr, La Jolla, CA 92093, United States

**Keywords:** bacteriophage, experimental evolution, key innovation, protein evolution

## Abstract

How viruses evolve to expand their host range is a major question with implications for predicting the next pandemic. Gain-of-function experiments have revealed that host-range expansions can occur through relatively few mutations in viral receptor-binding proteins, and the search for molecular mechanisms that explain such expansions is underway. Previous research on expansions of receptor use in bacteriophage λ has shown that mutations that destabilize λ’s receptor-binding protein cause it to fold into new conformations that can utilize novel receptors but have weakened thermostability. These observations led us to hypothesize that other viruses may take similar paths to expand their host range. Here, we find support for our hypothesis by studying another virus, bacteriophage 21 (Φ21), which evolves to use two new host receptors within 2 weeks of laboratory evolution. By measuring the thermodynamic stability of Φ21 and its descendants, we show that as Φ21 evolves to use new receptors and expands its host range, it becomes less stable and produces viral particles that are genetically identical but vary in their thermostabilities. Next, we show that this non-genetic heterogeneity between particles is directly associated with receptor use innovation, as phage particles with more derived receptor-use capabilities are more unstable and decay faster. Lastly, by manipulating the expression of protein chaperones during Φ21 infection, we demonstrate that heterogeneity in receptor use of phage particles arises during protein folding. Altogether, our results provide support for the hypothesis that viruses can evolve new receptor-use tropisms through mutations that destabilize the receptor-binding protein and produce multiple protein conformers.

## Introduction

As we push through the fourth year of a global pandemic, many researchers have turned their attention to understanding the risk factors and processes involved in potentiating pandemics (Franch-Pardo et al. 2020). A critical step in the initiation of a pandemic occurs when non-human viruses gain the capacity to recognize and bind to human cells (Ito et al. 1998). Often, this occurs through mutations in the viral receptor-binding protein (RBP), which allows them to attach to molecules on the surface of new host cells (Curiel 1999; Grimm et al. 2008, Meyer et al. 2012, Longdon et al. 2014). Since the beginning of the SARS-CoV-2 pandemic, there has been renewed interest in (i) understanding the types of evolutionary and molecular processes that lead to changes in RBPs tropism and (ii) determining whether there are general processes by which these molecular innovations evolve.

The evolution of novel functions in viral RBP can be informed by the broader understanding of gene evolution. The prevailing paradigm suggests that gene duplication, followed by subsequent evolution driven by genetic drift or natural selection, is a key mechanism for the emergence of new functions (Ohno 1970). Notably, there are two well-documented cases of viruses expanding their host range through duplication of their host-recognition genes. Specifically, duplication of the tail fiber gene in bacteriophage K1-5 has been shown to enable infection of multiple hosts, while duplication of a tail fiber domain in T4 bacteriophage has allowed it to broaden its host range (Tétart et al. 1996, Scholl et al. 2001).

Gene duplication as a starting point for RBP innovation may not be a general mechanism for viral evolution given that viral genomes are often constrained by the pressure to be compact (Belshaw et al. 2007). Additionally, the presence of overlapping or overprinted genes (Pavesi 2021), as well as structural constraints associated with the mechanical forces of displaying multiple distinct RBPs, may further restrict the feasibility of gene duplication (Lavillette et al. 2001). An alternative mechanism for exploring new functions is through folding heterogeneity, where a single peptide can evolve to exhibit flexibility or adopt multiple distinct conformations (Sikosek and Chan 2014). This concept of evolutionary innovation through nongenetic phenotypic heterogeneity was first proposed by C. H. Waddington over 80 years ago to explain fruit fly evolution (Waddington 1942). Recently, this idea has gained significant attention and has been implicated in driving evolutionary innovations in various systems, including protein evolution (Van Buskirk and Steiner 2009, Levis and Pfennig 2019, Sakuma et al. 2023).

The potential for folding heterogeneity to facilitate the evolution of new functions in viral RBPs has been observed in a series of studies on the evolution and host range expansion of bacteriophage λ (Petrie et al. 2018). When λ is cocultured in the laboratory with its host, *Escherichia coli*, λ rapidly and repeatedly evolves the use of a new receptor, OmpF, in addition to its original receptor, LamB, by acquiring four or more mutations in its RBP called J (Meyer et al. 2012). Mutations in the J protein typically evolve in two distinct regions. One set of mutations localizes to loops situated at the interface of the receptor-binding protein (RBP)-receptor interaction, whereas a second set occurs adjacent to the receptor-binding domain, where three J protein monomers form a trimeric structure (Strobel et al. 2022, 2024). Genetic experiments have demonstrated that loop mutations alone are insufficient to confer functionality on OmpF, and that the second set of mutations, which do not appear to directly interact with receptors, are also required for this purpose (Maddamsetti et al. 2018). Further studies have revealed that the second set of mutations induce thermodynamic instability and allow the J protein to adopt alternative conformations (Strobel et al. 2022, 2024). Detailed analyses of a specific J mutation show that it disrupts trans-monomer hydrogen bonding, thereby weakening trimer formation and contributing to the observed instability (Strobel et al. 2024). Notably, this instability increases the flexibility and dynamics of the J protein and enables it to adopt multiple distinct conformations (Petrie et al. 2018, Strobel et al. 2024). Some of these conformations are highly unstable and can interact with OmpF, while others are stable and reliant on LamB. Collectively, these findings suggest that λ phage evolves to utilize a new receptor through the acquisition of structurally destabilizing mutations in its RBP, which enable a single genotype to produce multiple phenotypes, some of which interact with the novel outer membrane molecule, OmpF.

The applicability of this mechanism to other viruses remains an open question. To address this, we investigated phage Φ21, a virus related to λ phage, which shares homologous essential genes but varies in gene content with respect to non-essential host virulence factors (Feiss et al. 2022). Notably, Φ21’s RBP, also known as J, exhibits 94% nucleotide identity with λ’s J, overall providing a unique evolutionary starting point to test this hypothesis (Ye et al. 2006). Recently, we discovered that Φ21 evolves to utilize two new receptors when co-cultured with its host, *E. coli* (Borin et al. 2023). This co-culture triggers a coevolutionary arms race, where *E. coli* evolves mutations that suppress the phage receptor LamB, prompting the phage to evolve a counter-defense strategy. Specifically, a single mutation in Φ21’s RBP enables the virion to recognize a new outer membrane protein, OmpC. In response, *E. coli* evolves additional mutations within *ompC*, which Φ21 counters by acquiring mutations in J that allow it to utilize a third receptor, OmpF. Ultimately, evolved Φ21 isolates can independently recognize and utilize all three receptors.

Here, we investigate whether Φ21 evolves new receptor use through the same molecular mechanism as λ. We confirmed that the Φ21 RBP mutations are destabilizing, likely by interfering with J-trimer formation, as they do in λ. Additionally, the J mutations induce the production of nongenetic phenotypic heterogeneity: isogenic stocks of evolved Φ21 possess a variety of particles with distinct phenotypes, including some that are thermodynamically stable and reliant on LamB, while others vary in their stabilities and use of OmpC and OmpF.

Beyond demonstrating the applicability of our molecular model to a novel virus, we also experimentally tested two key predictions derived from the model. Firstly, we hypothesized that if phage phenotypic heterogeneity arises during protein folding, then overexpressing host-cell chaperones would modulate the resulting phenotypic heterogeneity. Our findings support this prediction. Secondly, leveraging the fact that Φ21 has evolved to utilize two novel receptors, we predicted a correlation between phage genotype instability and receptor usage. However, our results did not reveal a significant relationship, suggesting that more instability does not necessarily lead to broader functionality.

## Results

### Decay dynamics of the Φ21 ancestor and triple receptor-using ΦD9

We began by studying ΦD9, a phage derived from Φ21 which was isolated on Day 9 of a previous coevolution experiment (Borin et al. 2023). ΦD9 was the earliest phage isolate demonstrating an evolved ability to use two new receptors, OmpC and OmpF, in addition to its native receptor LamB, through the acquisition of six mutations (four mutations in its RBP, one in a separate tail fiber protein, and one in a non-tail structural gene). As observed in evolved λ phages, the acquisition of destabilizing mutations in RBPs can lead to a rapid loss of viral infectivity over time, which can be quantified by monitoring the decline in viable phage particles (plaque-forming units; PFUs) in phage lysate without host cells. Although the exact reason why J instability leads to phage mortality is unclear, we hypothesize that unstable J proteins are more prone to misfiring, given their crucial role in initiating infection and ejecting DNA (Ge and Wang 2024).

We tested whether mutations in ΦD9 caused a decrease in stability relative to its ancestor Φ21. Additionally, we examined whether ΦD9 displays multiphasic decay kinetics, as observed in λ. In contrast to the typical monophasic exponential decay exhibited by most phages (De Paepe et al. 2006), phages that produce heterogeneous particles can exhibit multiphasic decay patterns (Petrie et al. 2018). Our underlying hypothesis is that proteins capable of adopting multiple conformations may exhibit distinct thermodynamic properties, resulting in varying rates of spontaneous inactivation of infectivity (i.e. decay). Therefore, multiphasic decay patterns can be used as a rudimentary indicator for whether an isogenic population of phages possess multiple phenotypes.

**Figure 1.**
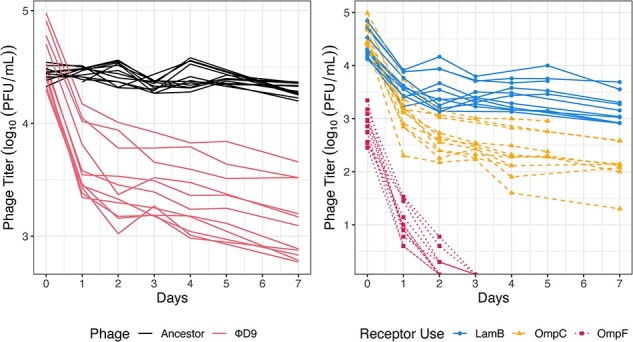
Phage Φ21 evolves multiphasic decay concomitant with host receptor-use expansion. Panel A: Titer of Φ21 ancestor (black) and evolved triple-receptor using phage ΦD9 (pink) decaying over 7 days. A monophasic decay model was the best fit for the ancestor and a biphasic decay model was the best fit for ΦD9 (Table S1). Panel B: Titer of subpopulations of phage particles that can infect using different host receptors. Lines represent individual replicates. Phage particle subpopulations with different receptor-use tropisms had significantly different initial decay rates (ANOVA, *P* < .0001; Tukey’s HSD, *P* < .01).

ΦD9 decayed significantly faster than its ancestor (*P* < .0001, T-test comparing initial decay rates; Fig. 1a). In addition, we found that the ancestor appears to be monomorphic because it decays at a single exponential rate, whereas ΦD9 was best fit by a biphasic exponential decay model, suggesting that the isogenic phage lysate was comprised of at least two phage subpopulations with different decay rates (Fig. 1a; Supplementary Material, Fig. S1, Table S1).

Next, we tested whether the biphasic decay pattern observed in ΦD9 is related to the differential usage of distinct receptors by measuring the rate at which the isogenic phage population loses its ability to utilize these receptors. If all phage particles exhibit uniform receptor affinity, we would expect a uniform loss of receptor binding capacity. However, if phage particles with varying receptor affinities possess distinct thermodynamic stabilities, then we would anticipate variable loss of affinities across the different receptors. This test can be conducted by repeating the previous decay experiment and additionally plating on three cell types that each expresses only one of the three receptors (LamB, OmpC, or OmpF).

In support of our hypothesis that the observed heterogeneity in particle decay rates underlies functional heterogeneity in receptor use, we found that subpopulations with different receptor preferences decayed at different rates. Particles that can use the native receptor (LamB) were the most stable, followed by particles that use the second receptor (OmpC), and the least stable were particles that use the third receptor, OmpF (Fig. 1b; ANOVA, *P* < .0001; Tukey’s HSD, *P* < .01). Interestingly, this reflects the order in which the new receptor-use functions evolved. The three significantly different decay rates measured from isogenic stocks of ΦD9 suggest that the isogenic ΦD9 culture has at least three different protein conformations with three different thermostabilities (Fig. 1b). The different thermostabilities associated with different receptor-using phage particles support that Φ21 evolved to use novel receptors by producing a multimorphic population of particles in accordance with our hypothesis. This result provides a first non-λ example of RBP gain of function achieved through destabilizing mutations that cause non-genetic phenotypic heterogeneity.

### Link between protein folding and the production of non-genetic phenotypic heterogeneity

We hypothesized that heterogeneity in phage particles arises due to stochasticity in the folding of RBPs in the host cell. To test our hypothesis, we altered the concentration of protein-folding chaperones in the host during phage infection by manipulating expression of *rpoH* and then measuring the relative ratio of particles able to use LamB versus OmpC and OmpF. This was achieved by plating subsamples of the phage populations grown on host with and without *rpoH* induction. Subsamples were split and plated on lawns of cells that only express LamB, OmpC, or OmpF, and the number of PFUs on each host was compared to the PFUs that formed on lawns of wild-type cells that express all three receptors. The ratio of PFUs is called Efficiency of Plaquing (EOP). Our prediction was that EOP should increase on the LamB-only cells when *rpoH* was induced, since the chaperones could assist J folding by allowing J to uncover lower energy conformations, resulting in an increased number of stable LamB-reliant particles.

**Figure 2.**
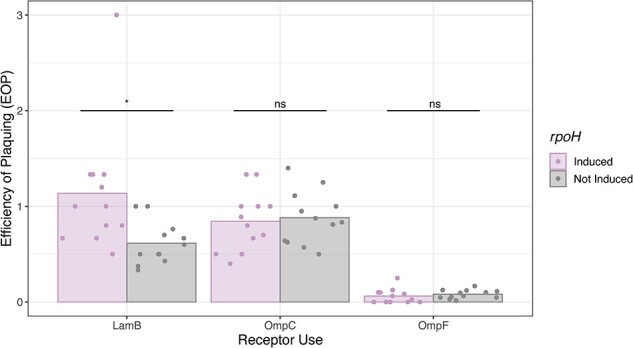
Increased expression of chaperone proteins (*rpoH* induction) shifts particle formation towards stable, LamB-using variants. Efficiency of plaquing (EOP) was calculated as the relative titer of phages on each receptor (i.e. single-receptor hosts) compared to on all receptors (i.e. wild type). Significant differences in EOP treatments for each receptor are indicated above bars (*, *P* < .05; ns, not significant; T-test; n = 12 replicates per treatment).

In support of our hypothesis, we found that when *rpoH* was induced, the EOP on the LamB receptor was significantly higher than in cells without *rpoH* induction (T-test, *P* = .02) (Fig. 2). One rpoH-induced datapoint showed a considerably higher EOP on LamB than other replicates, calling into question whether the difference observed was driven by an outlier. However, removing this point strengthens the statistical significance of our results (T-test, *P* = .005). Altogether, by manipulating the kinetics of protein folding through manipulating chaperone expression and showing it alters the phenotypic heterogeneity, we demonstrated an important link between protein folding stochasticity and the creation of the heterogeneity. These experiments provide mechanistic support for our hypothesis that heterogeneity in RBP folding results in the formation of a range of conformers with different receptor-use functionalities.

### Stability changes during Φ21 coevolution

**Figure 3.**
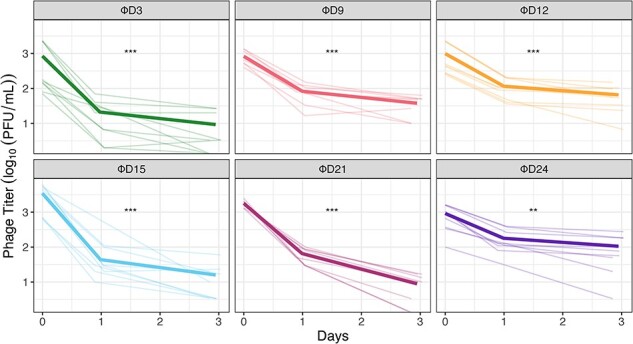
Evolved Φ21 isolates with different host range breadths all demonstrate multiphasic decay. Phage titers were enumerated over 3 days of decay at 37˚C. Bold lines represent the mean and faint lines represent independent replicates (n = 9). Titer was measured by plating phages and enumerating plaques on soft agar lawns of WT cells (for ΦD24, which only uses the OmpF receptor, we used lawns of ∆LamB ∆OmpC cells). Differences in decay rates between days 0–24 h and 24–72 h are indicated by asterisks (**, *P* < .01; ***, *P* < .001; paired T-test).

Thus far, we have focused on comparing the Φ21 ancestor and a derived variant (ΦD9) that evolved to use three receptors. For the rest of the study, we explore whether other evolved variants demonstrated similar decay patterns. We studied five new phages isolated from different timepoints or with unique receptor usages (Table 1). Previously in this manuscript we described the evolution of receptor-use expansion to OmpC and OmpF. Interestingly, this is only the first phase of receptor-use changes during coevolution between Φ21 and *E. coli*. As phages continue to coevolve with their hosts, they refine their receptor usage, shifting towards specialized interactions with OmpF, the sole remaining receptor expressed by *E. coli* at latter days of the coevolution experiment (Borin et al. 2023). Consequently, Φ21 gradually loses infectivity on LamB and OmpC. This evolutionary sequence of evolved Φ21 genotypes presents an opportunity to investigate the relationship between the stability of phage genotypes with different characteristics, while controlling for different receptor-use profiles and duration of laboratory coevolution. We measured the decay of these new evolved phages over 3 days (sampling at time points 0, 24, and 72 h) by enumerating the total phage density by plating on wild-type (WT) cells (Fig. 3). These timepoints were sufficient to characterize the overall differences in decay rates, as well as to test whether strains exhibited multiphasic decay by comparing decay rates calculated from 0–24 h and 24–72 h. To ensure that these results were comparable with previous experiments, we also included ΦD9. We found that all evolved phages were less stable than the ancestor and they all show patterns that suggest biphasic decay (Fig. 3; paired T-test of difference in decay rate from 0–24 h and 24–72 h. ΦD3, *P* < .001; ΦD9, *P* < .0001; ΦD12, *P* < .0001; ΦD15, *P* < .0001; ΦD21, *P* < .0001; and ΦD24, *P* < .005).

**Table 1.** Relevant characteristics of phage genotypes included in this study. All phages are descendants of the Φ21 ancestor and were isolated from the same population. Additional information on these phages is reported by Borin et al. (2023).

Phage	Receptor use	No. of receptors	No. of RBP mutations	Days coevolved
Φ21 (ancestor)	L	1	0	0
ΦD3	LC	2	1	3
ΦD9	LCF	3	4	9
ΦD12	LCF	3	5	12
ΦD15	CF	2	7	15
ΦD21	LF	2	11	21
ΦD24	F	1	10	24

**Figure 4.**
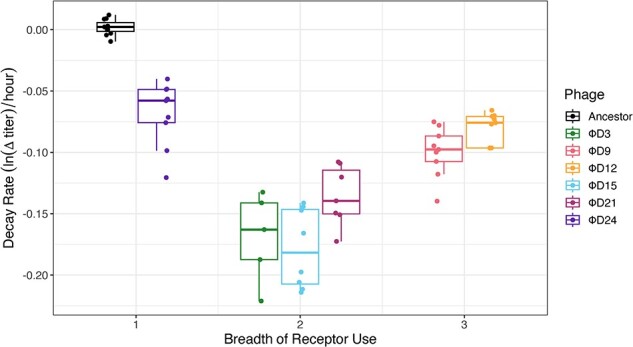
Relationship between breadth of host-receptor use and decay rate of seven phage genotypes with different receptor-use capabilities. Dual receptor-using phages were the least stable demonstrating a lack of relationship between receptor-breadth and instability.

Next, we explored how the evolution of destabilization relates to different characteristics to better understand the drivers of this key trait. First, we tested whether there was a relationship between the number of receptors a phage can use (receptor breadth) and how unstable it is (Fig. 4). We expected that there may be an inverse relationship between thermostability and functional heterogeneity since less stable proteins should be able to accommodate even more confirmations and receptor interactions. We found that, by Day 3, there is destabilization and the production of phenotypic heterogeneity. Yet, beyond Day 3, the relationship between receptor use breadth and stability was not significant (ANOVA, *P* > .05). Although Φ21 becomes unstable when it evolves to use the second receptor (OmpC, from ancestor to ΦD3), it then increases in stability when it evolves to use a third receptor (ΦD3 to ΦD9). Then it loses stability again as it evolves in two separate lineages that lose either LamB (ΦD12 to ΦD15) or OmpC (ΦD12 to ΦD21). At later timepoints, Φ21 evolves to specialize on OmpF, becoming the most stable of the evolved phage isolates, yet it is still less stable than the ancestor, which specializes on the LamB receptor. Altogether, these results suggest that only a certain threshold level of instability is required to produce confirmational heterogeneity required to explore new function, and that there is no monotonic relationship between instability and heterogeneity that drives increased receptor-use breadth.

**Figure 5.**
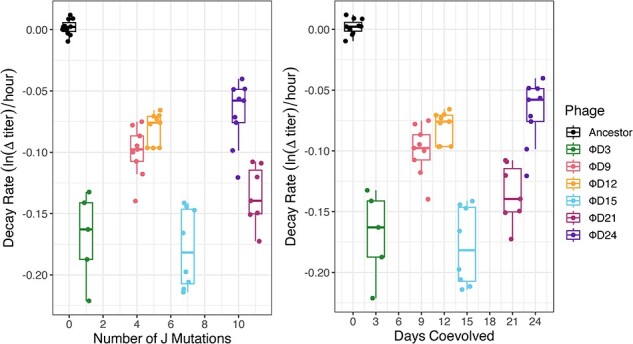
Relationship between phage decay rate and the number of evolved J mutations (Panel A) or days of coevolution (Panel B). The earliest phage to evolve (ΦD3) with only a single mutation is not significantly more stable than the most unstable phage (ΦD15).

We also tested whether the number of evolved J mutations or the duration of coevolutionary time was related to stability through linear regression analyses. In both regressions, we found that statistical significance was contingent on inclusion of the Φ21 ancestor (Fig. 5a; linear model, decay rate ∼ number of J mutations, with Φ21 ancestor *P* < .01 & negative slope, without Φ21 ancestor *P* > .05 & positive slope) (Fig. 5b; linear model, decay rate ∼ days coevolved, with Φ21 ancestor *P* < .01 & negative slope, without Φ21 ancestor *P* < .05 & positive slope). Additionally, removing the ancestor from analyses flipped the direction of the linear relationship. We suspect that the absence of a biologically meaningful relationship between stability and calendar time and evolutionary divergence may be due to the conflicting tension between selection to produce heterogeneity and explore new functions and costs associated with the destabilization. This idea has been demonstrated in influenza RBP (hemagglutinin) evolution, where viruses adapt along a thermodynamic boundary through protein destabilizing and compensatory mutations (Gong et al. 2013).

### Protein structure predictions

**Figure 6.**
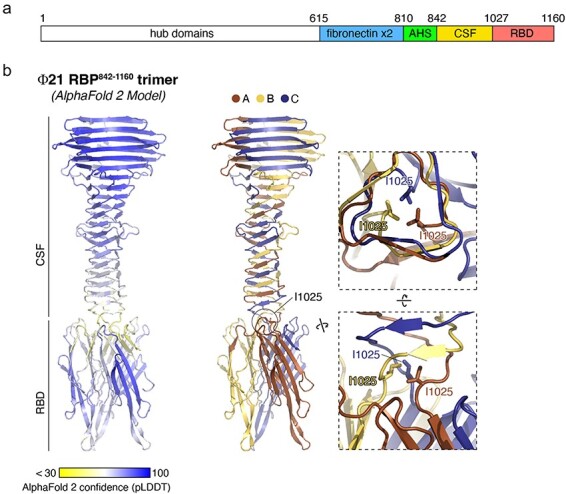
Structure prediction of the Φ21 RBP C-terminal domain trimer. Panel A: Domain schematic of the Φ21 RBP predicted by sequence alignments and structure predictions. Panel B: Left: Predicted structure of the Φ21 RBP central straight fiber domain (CSF) and receptor-binding domain (RBD), colored by AlphaFold 2 confidence (pLDDT) scores. Center: Predicted structure of the Φ21 RBP CSF and RBD with individual protomers colored brown, yellow, and blue. The position of residue I1025 is highlighted. Right: Two closeup views of three I1025 residues making hydrophobic interactions at the central trimer interface of Φ21 RBP.

The earliest isolate from the experiment (ΦD3) was sampled after only 3 days of coevolution and had a single mutation in its genome located in the RBP (Table 1). This strain gained the ability to infect using the OmpC receptor, was one of the least stable variants studied, and was bistable, demonstrating that the first evolutionary step taken by the phage simultaneously destabilized the RBP, generated phenotypic heterogeneity, and conferred new host receptor use. This mutation caused a shift from isoleucine to threonine at position 1025, just before the receptor-binding domain is predicted to start (Fig. 6). Notably, this mutation is in the same region as destabilizing mutations in λ’s RBP that are essential for gain of function on its new receptor, OmpF (Strobel et al. 2022). Recently, Strobel et al. (2024) demonstrated that, in λ, amino acids in this region of the RBP are involved in trimer formation and substitutions can destabilize hydrogen bonds between trimers, interfere with trimer formation, and cause both instability and gain of function (Strobel et al. 2024). Therefore, we hypothesized that the I1025T mutation may have similar effects on Φ21. We tested our hypothesis by modeling Φ21’s RBP structure and trimer formation. We found that residues I1025 and V1026 are located in the center of the tail fiber tube at its distal end, just before the receptor binding domains form (Fig. 6). These residues likely stabilize the interaction between the three protomers’ receptor-binding domains and mutating them may alter the stability of these domains’ associations. Given I1025’s precarious location at the junction between domains, weakened interactions from mutation of this residue could allow the receptor-binding domains more conformational freedom to dynamically bind receptors.

## Discussion

Viruses often evolve to infect new hosts by modifying their ability to interact with new host receptors (Tétart et al. 1996, Yehl et al. 2019, Boon et al. 2020). Previous work on phage λ demonstrates that viruses can evolve host receptor expansion through the creation of multimorphic intermediate phenotypes that allow the exploration of novel host tropisms. Here, we show that a new phage, Φ21, takes a similar path as it evolves to use two new receptors. First, we demonstrate that the receptor binding protein (RBP) of ΦD9, a descendant of Φ21, loses thermostability as it broadens its receptor range and gains the ability to use two new receptors. As in evolved strains of phage λ, the evolved ΦD9 genotype produces a heterogeneous population of phage particles comprised of RBPs with different protein folds that have different thermostabilities and concomitant host receptor tropisms. To test the link between protein folding and host receptor use (function), we manipulated the presence of host chaperone proteins that assist disordered proteins in achieving more stable conformations. These experiments showed that when chaperones were present, phage proteins were more likely to fold into conformations with greater stability that also have a stronger affinity for the native receptor, LamB. Finally, by measuring the decay patterns of five additional phage genotypes, with different receptor-use preferences and accumulated RBP mutations, we showed that heterogeneity in phage particles was maintained throughout Φ21’s evolution as its receptor tropism changed to produce a diverse array of receptor-use types. Lastly, we proposed a likely cause of the instability, a mutation that weakens trimer formation at the interface between CSF and RBD domains.

Consistent with previous research conducted on λ, we see that shifts in viral RBP tropism through destabilizing mutations allow for the evolution of new functions while still conferring the ability to infect through the native receptor. Previous studies have referred to the multi-receptor using phage particles as producing non-genetic phenotypic heterogeneity, and this non-genetic variation has been found to drive molecular innovation (Petrie et al. 2018). Here, we have shown that this process of viral receptor binding protein variation readily evolves in Φ21 as well. Moreover, by altering the heterogeneity of phage receptor-use types by manipulating of host protein-folding chaperones, we have expanded our understanding of how this evolutionary process occurs.

This research has important implications for understanding features of protein evolvability. The traditional view is that more stable proteins are more likely to innovate, since they can accommodate more adaptive mutations (mutational robustness) that incur a pleiotropic cost of destabilization (Wang et al. 2002, Bloom et al. 2006, Studer et al. 2014). However, there is a growing realization that the relationship between protein mutational robustness and evolvability may be more nuanced. In some cases, less-stable proteins may be more evolvable if the instability that underlies the formation of phenotypic heterogeneity can be used for functional innovation (Tokuriki and Tawfik 2009, Sikosek and Chan 2014, Dellus-Gur et al. 2015). In support of the hypothesis that unstable proteins could enable–rather than hinder–the evolution of new functions, we find that the very first mutation Φ21 that evolves destabilizes the phage particle and produces heterogeneous phage particles that allow it to use the novel receptor, OmpC. Even though this observation is not a direct test of this hypothesis, our results support the fact that destabilized proteins can be more evolvable than their structurally rigid counterparts (see Strobel et al. 2022 for a direct test with λ’s RBP). This role of instability promoting protein evolvability does not appear to be limited to viral RBP, as a similar phenomenon has been observed in the evolution of new alkaline phosphatase activity in *E. coli* (Sakuma et al. 2023).

Two laboratory studies have shown that RBPs can evolve to recognize new receptors by accumulating destabilizing mutations that introduce variability in protein folding. However, it remains unclear whether viruses in natural environments use this same mechanism to evolve, or if environmental pressures prevent them from doing so. Mathematical modeling studies, based on data from λ genotypes’ ability to bind to LamB and OmpF, suggest that natural selection would favor RBP variants that exhibit high levels of phenotypic variation because they can access a variety of cell types (Petrie et al. 2018). This implies that such proteins may be common in nature. However, these models did not consider the tradeoff between the benefits of heterogeneity and the costs of protein instability. The cost of instability likely varies depending on the environment. In environments with high host density, the tradeoff is minimal, and viruses can likely deploy this mechanism of RBP evolution. In contrast, if viruses need to survive for extended periods, the cost of instability becomes significant, as up to 95% of viral particles may spontaneously decay within a day. In these environments, the evolution of RBP variants with high heterogeneity may be constrained.

There is some direct evidence that viruses deploy this mechanism during their evolution. Analyses of natural λ J-related protein sequences have revealed heightened rates of sequence evolution at the same nucleotide positions that evolve during laboratory experiments, suggesting that similar evolutionary processes are at play in natural ecosystems (Maddamsetti et al. 2018). Furthermore, a systematic analysis of viral proteins has shown that they are generally more disordered and prone to thermodynamic instability than their cellular counterparts (Kramer et al. 2006), which could make phenotypic heterogeneity, and therefore evolutionary innovations, more accessible. Taken together, these observations suggest that destabilization and the production of disordered proteins may be a more common path of viral evolution than previously appreciated.

## Methods

### Strains

All phage isolates used in this study are descendants of a strictly lytic version of Φ21 (GenBank: OL657228; Feiss et al. 2022) and were first reported by Borin et al. (2023). Relevant information about the phages used in this study is provided in Table 1. For bacterial hosts, *E. coli* K-12 strain BW25113 (WT) and related gene knockout mutants were used for culturing and plating phages (Baba et al. 2006; receptor double- and triple-receptor knockout strains described by Borin et al. 2023). For experiments where we manipulated host protein-folding chaperones, we used ASKA strain JW3426 [National BioResource Project (NIG, Japan)], which has gene *rpoH* (sigma32) provided on the pCA24N plasmid under the control of an IPTG-inducible T5-lac promoter (Kitagawa et al. 2005).

### Phage ΦD9 decay experiment

To investigate whether Φ21 evolved phenotypic heterogeneity as it expanded the range of receptors it could use, we compared the decay patterns of the Φ21 ancestor with ΦD9. Phage decay was measured by first generating replicate isogenic phage lysates. Scrapes (∼2 uL) from a frozen preserved isogenic phage stock were inoculated into replicate tubes containing 4 mL Tris-LB and ∼10^7^ host cells (recipe by Borin et al. 2023). ΦD9 was grown with ΔLamB ΔOmpC host cells in order to maintain its ability to use the OmpF receptor. The Φ21 ancestor was grown with ΔOmpC cells (KEIO strain JW2203) to prevent its evolution to use the OmpC receptor. After incubating co-cultures for 4 h, shaking at 37˚C, phages were extracted using 0.2-μm filters.

Phage lysates were then studied immediately to limit the decay of phages. Phages were first diluted in Tris-LB and ∼3x10^5^ particles were aliquoted into replicate glass tubes and incubated at 37˚C without shaking to reduce evaporation. Initially and every day for 7 days, 100 μL was removed from each tube and inoculated into molten (∼55˚C) 0.7% w/w LB soft agar (recipe by Borin et al. 2023) infused with ∼10^8^ WT cells, swirled onto LB agar plates, and incubated overnight at 37˚C. After incubation, plaques were enumerated. We used WT because it expresses all three relevant host receptors and provides an estimate of all phage particles. The decay experiment was performed on a total of 12 replicates conducted in two separate batches. To compare the overall decay rate of the Φ21 ancestor and the evolved ΦD9 phage, we computed the exponential decay rate of each replicate between Day 0 and Day 7 (Equation 1) and compared the rates using a T-test, implemented in R (version 4.1.1; R Core Team 2016).

### Population decay model fitting

To study whether decay patterns of phages were suggestive of a single, monomorphic population of phage particles or a heterogeneous population comprised of particles with different decay rates, we used linear regression and model fitting (Fig. S1, Table S1). We fit models that were representative of populations containing a single decay rate (Eq. 1; monophasic), heterogeneous populations containing two (Eq. 2; biphasic) or three (Eq. 3; triphasic) discrete decay rates, and a model where decay rate changed continuously over time (Eq. 4, derived from Eq. 5) (Petrie et al. 2018). After fitting each model to the observed data, we calculated the Akaike Information Criterion (AIC) and log-likelihood to select the most supported model for our data (e.g. lowest AIC, a composite of fit and model complexity; significant *P*-value in model comparison, significant increase in fit).


(1) $$\ln {N_t} = \ln \left( {{N_0}{e^{ - rt}}} \right)$$

(2) $${\mathrm{ln\,}}{N_t} = \ln \left( {\left( {\left( {\frac{a}{{a + b}}} \right) \times {N_0}{e^{ - {r_a}t}}} \right) + \left( {\left( {\frac{b}{{a + b}}} \right) \times {N_0}{e^{ - {r_b}t}}} \right)} \right)\,$$

(3) $$\begin{aligned}\ln {N_t} = & \ln \big(\left( {\left( {\frac{a}{{a + b + c}}} \right) \times {N_0}{e^{ - {r_a}t}}} \right) + \left( {\left( {\frac{b}{{a + b + c}}} \right) \times {N_0}{e^{ - {r_b}t}}} \right.) \\& \nonumber + (\left.{\left( {\frac{c}{{a + b + c}}} \right) \times {N_0}{e^{ - {r_c}t}}} \right)\big) \end{aligned}$$

(4) $$\ln {N_t} = \ln \left( {{{\left( {\left( {a \times \left( {r - 1} \right) \times t} \right) + \left( {{N_0}^{\left( {1 - r} \right)}} \right)} \right)}^{\frac{1}{{\left( {1 - r} \right)}}}}} \right)$$

(5) $$\frac{{dN}}{{dt}} = \, - r{N^\delta }$$

In order to test whether the decay of evolved ΦD9 is linked to subpopulations of phage particles with different receptor-use tropisms, we repeated the 7-day decay experiment described above except that at each timepoint we plated ΦD9 onto a suite of double-receptor knockout hosts (ΔOmpC ΔOmpF, ΔLamB ΔOmpF, and ΔLamB ΔOmpC) to enumerate the particles that remained viable and could infect through each different receptor. We then calculated the proportion of phage particles that could use each receptor by calculating the efficiency of plaquing (EOP) by dividing the density of phage particles that could use a given receptor by the total density of phage particles (determined by enumerating the phage lysate on WT cells which present all three receptors). To compare the decay rate of each subpopulation that could infect a different receptor, we calculated initial exponential decay rates (between 0 and 48 h) and then analyzed these rates using ANOVA and Tukey’s HSD tests, implemented in R.

### Protein folding and host receptor shift experiment

To test whether there is a link between the heterogeneity of phage receptor use and phage protein folding, we modified the expression of host heat-shock chaperone proteins during infection to test if this would alter phage protein folding and produce phages with different receptor-use preferences. To do this, we grew *E. coli* cultures by inoculating a colony of JW3426 grown on an agar plate (LB agar with 0.001 molar chloramphenicol and 0.8 molar IPTG) into tubes containing 4 mL LB supplemented with the same concentrations of chloramphenicol and IPTG, and incubated the cultures overnight, shaking at 30˚C. The next day, cells were washed four times by pelleting cells via centrifugation (21 130 × *g* for 2 min) and resuspending them in Tris-LB. After washing, ∼10^7^ cells were inoculated into 4 mL LB with chloramphenicol but without IPTG and incubated overnight, shaking at 37˚C. After incubation, cells were washed again, as described above, to remove any remaining IPTG. Finally, ∼10^7^ cells were inoculated into 12 replicate tubes containing 4 mL Tris-LB supplemented with 0.1 molar chloramphenicol and ∼10^4^ particles of ΦD9. Half of the replicates were also supplemented with 0.001 molar IPTG and half were not (n = 6 replicates in each treatment). Cultures were incubated at 37˚C, allowing phages to replicate on each of the hosts in each treatment, and after 2 h phages were extracted using 0.2 μm filters, serially diluted, spotted on LB agar plates infused with WT or our collection of double receptor knockout hosts, and incubated overnight at 37˚C. The next day, we enumerated plaques and calculated the EOP. The entire experiment was conducted twice, and no day effects were observed (*P* > .05). To determine whether there were differences in host receptor preferences between phages grown on host cells with or without *rpoH* expressed, we compared the EOP between treatments on each receptor using T-tests, implemented in R.

### Decay of evolved phages with diverse receptor-use tropisms

During coevolution with its *E. coli* host, Φ21 diversifies into phage strains with different receptor use capabilities (conferred by the acquisition of different numbers of mutations in the RBP, called J). To investigate whether and when receptor use expansion and contraction was associated with protein destabilization and the creation of phenotypic heterogeneity, we measured the decay of representative phage isolates with different receptor use capabilities (isolated from the earliest day on which that receptor use type was observed). Decay experiments were conducted as described above, except with the following changes: Phages ΦD3, ΦD9, ΦD12, ΦD15, ΦD21, ΦD24 were grown up from frozen preserved stocks with WT, ∆LamB ∆OmpC, WT, ∆OmpC, ∆LamB, or ∆LamB ∆OmpC hosts, respectively. We chose hosts that produced a high density of phage particles while also maintaining the receptor use range of the phage isolate. Experiments were conducted for 3 days, which encompassed >93% of the decay that was observed in previous experiments on ΦD9. To test whether protein stability was related to receptor use breadth, we fit linear models comparing the initial exponential decay rate (0–24 h) and the number of receptors each phage strain could use. Similarly, we compared phage stability with the number of evolved mutations in the phage’s RBP, as well as against the number of days the phage isolate had evolved.

### RBP structure predictions

To study how a key RBP mutation (I1025T) affects Φ21 stability, we used AlphaFold 2 to predict the structure of the C-terminal 319 amino acids of Φ21 RBP that includes the central straight fiber domain (CSF) and receptor-binding domain (RBD) of the protein, as a homotrimer. We used the publicly available ColabFold-mmseqs2 implementation (Mirdita et al. 2022) of AlphaFold-multimer (Jumper et al. 2021, Evans et al. 2022). We used PyMOL (Schrodinger Inc.) and Chimera (Pettersen et al. 2004) to pinpoint where the mutation occurred and to visualize how it could affect protein stability.

## Supplementary Material

veae049_Supp

## Data Availability

The data underlying this article are available at https://doi.org/10.5061/dryad.wwpzgmsrw.
