# Supplementary material for: Bacteriophage Φ21’s receptor-binding protein evolves new functions through destabilizing mutations that generate non-genetic phenotypic heterogeneity
Source: Virus Evol. 2024 Jul 11;10(1):veae049. doi: 10.1093/ve/veae049 (PMC11336670; doi:10.1093/ve/veae049)
Supplement: veae049_Supp [file veae049_supp.zip › suppl_data/Gerbino 2024 VirEvo Supplementary Materials.docx]

**Supplementary Materials**

Population decay model selection

To assess whether populations of ancestral phage Φ21 and evolved phage ΦD9 demonstrate monophasic, biphasic, triphasic or continuous decay patterns, we fit 4 models (Eq. 1-4) to each population-level decay curve using the R gslnls package (Fig. S1). To differentiate between nested models (Eq. 1-3), we performed log-likelihood ratio tests (LRT). To differentiate between non-nested models, we selected the model with the lowest Akaike Information Criterion (AIC). For the Φ21 ancestor, Model 1 (e.g., monophasic decay, Eq. 1) was selected as the best fit model. The addition of parameters for bi-and tri-phasic decay did not significantly increase the variance explained in the population level decay (P>0.05, LRT). Further, the AIC of Model 1 is lower than that of Model 4, confirming the choice of monophasic decay as the best fit of these models to this data (Table S1). For ΦD9, Model 2 (e.g., biphasic decay, Eq. 2) was selected as the best fit model. Eq. 2 significantly improves the variance in population decay explained relative to Eq 1. (p<0.01, LRT); however, Eq. 3 does not significantly increase the goodness of fit over Eq. 2 (p=0.78, LRT). Additionally, the AIC of Model 2 is lower than that of Model 4, further supporting that ΦD9 demonstrates biphasic decay.


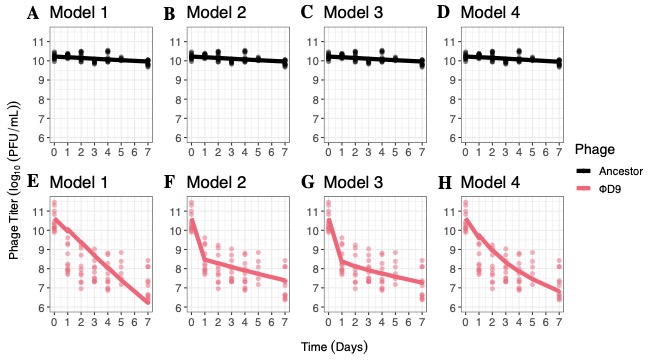


Figure S1. Exponential decay models indicate monophasic decay curves in the Φ21 ancestor and biphasic decay curves in the evolved ΦD9 phage. Models 1–4 were fitted based on equations 1–4 in the main text and represent one, two, or three population decay rates, or a continuously decaying model, respectively. Panels A–D: Models 1–4 fit to Φ21 ancestor decay over 7 days. Panels E–H: Models 1–4 fit to ΦD9 decay over 7 days. Model 1 was selected as the best fit to the decay pattern of the Φ21 ancestor over 7 days. Model 2, indicating biphasic decay, was the best fit model to the decay pattern of the evolved ΦD9 phage.

Table S1. Φ21 ancestor and ΦD9 decay patterns analyzed with Akaike Information Criterion (AIC) for model selection. Table denotes phage ID, model number, and its relevant AIC. The lowest AIC, determined through fit and model complexity, of each phage ID suggests the most supported model.

| Phage | Model Fitted | Akaike Information Criterion (AIC) |
| --- | --- | --- |
| Φ21 ancestor | Model 1 | -39.5649 |
| Φ21 ancestor | Model 2 | -33.5649 |
| Φ21 ancestor | Model 3 | -29.5649 |
| Φ21 ancestor | Model 4 | -38.52257 |
| ΦD9 | Model 1 | 228.5354 |
| ΦD9 | Model 2 | 153.2392 |
| ΦD9 | Model 3 | 157.7206 |
| ΦD9 | Model 4 | 198.7129 |
